# A Systems Biology Approach Uncovers Cellular Strategies Used by *Methylobacterium extorquens* AM1 During the Switch from Multi- to Single-Carbon Growth

**DOI:** 10.1371/journal.pone.0014091

**Published:** 2010-11-24

**Authors:** Elizabeth Skovran, Gregory J. Crowther, Xiaofeng Guo, Song Yang, Mary E. Lidstrom

**Affiliations:** 1 Department of Chemical Engineering, University of Washington, Seattle, Washington, United States of America; 2 Department of Microbiology, University of Washington, Seattle, Washington, United States of America; Texas A&M University, United States of America

## Abstract

**Background:**

When organisms experience environmental change, how does their metabolic network reset and adapt to the new condition? *Methylobacterium extorquens* is a bacterium capable of growth on both multi- and single-carbon compounds. These different modes of growth utilize dramatically different central metabolic pathways with limited pathway overlap.

**Methodology/Principal Findings:**

This study focused on the mechanisms of metabolic adaptation occurring during the transition from succinate growth (predicted to be energy-limited) to methanol growth (predicted to be reducing-power-limited), analyzing changes in carbon flux, gene expression, metabolites and enzymatic activities over time. Initially, cells experienced metabolic imbalance with excretion of metabolites, changes in nucleotide levels and cessation of cell growth. Though assimilatory pathways were induced rapidly, a transient block in carbon flow to biomass synthesis occurred, and enzymatic assays suggested methylene tetrahydrofolate dehydrogenase as one control point. This “downstream priming” mechanism ensures that significant carbon flux through these pathways does not occur until they are fully induced, precluding the buildup of toxic intermediates. Most metabolites that are required for growth on both carbon sources did not change significantly, even though transcripts and enzymatic activities required for their production changed radically, underscoring the concept of metabolic setpoints.

**Conclusions/Significance:**

This multi-level approach has resulted in new insights into the metabolic strategies carried out to effect this shift between two dramatically different modes of growth and identified a number of potential flux control and regulatory check points as a further step toward understanding metabolic adaptation and the cellular strategies employed to maintain metabolic setpoints.

## Introduction

*Methylobacterium extorquens* AM1 is a facultative methylotrophic bacterium capable of growth on single-carbon compounds such as methanol, methylamine and formate, and multicarbon compounds such as pyruvate and succinate [1]–[3]. In addition to their role in the global carbon cycle [4], methylotrophs are of interest for their potential in the biotechnological production of valued-added chemicals from methanol, which is an inexpensive and abundant source of carbon [5]. These organisms are ubiquitous in nature and are often associated with the leaf surfaces of plants where methanol is released when the stomata open in the morning [6]–[7]. To accommodate these bursts of methanol, methylotrophs must be poised to quickly adapt, capturing available methanol while preventing buildup of its subsequent toxic oxidation product, formaldehyde, as well as further downstream toxic metabolites such as glyoxylate and glycine [8]–[9]. Because of the high flux of toxic metabolites produced during methylotrophic growth, this metabolic mode of growth presents an interesting case study regarding the regulation of and balance between production and consumption of these toxic metabolic products.

*M. extorquens* AM1 has been studied for decades (reviewed in [10]) and is a model organism for understanding the metabolic components required for methylotrophic growth. The most broadly studied physiological condition has been the comparison of growth on methanol vs. succinate. When growing on multi-carbon substrates, *M. extorquens* AM1 uses pathways that are common to many heterotrophs, including the TCA cycle, the pentose-phosphate pathway, parts of anapleurotic pathways and gluconeogenesis, and an electron transport chain involving NADH dehydrogenase [1], [11]. In contrast, growth on single-carbon compounds involves specific metabolic pathways for both energy metabolism and assimilation, and requires over 100 additional gene products for central metabolism [10]. These two modes of growth are dramatically different, with methanol growth predicted to be limited by reducing power (NAD(P)H) while succinate growth is predicted to be limited by energy (ATP) [12]–[13].

Global approaches have been developed for *M. extorquens* AM1 to study multiple layers in the metabolic hierarchy including transcriptomic [14], metabolomic [15]–[17] and proteomic [18]–[19] approaches, as well as analysis of enzymes and fluxes [13], [20]–[21]. These individual studies have shown that the majority of genes known to function in C_1_ and multicarbon metabolism are differentially expressed and produced according to their function in these two different modes of growth. Most of the amino acids, biosynthetic precursors and intermediates involved in the serine cycle (the main methylotrophic assimilatory pathway) and the TCA cycle are at similar concentrations for methanol growth and succinate growth, while intermediates of the methylotrophy-specific ethylmalonyl-CoA (EMC) pathway are highly elevated in methanol cultures (up to 24-fold differences) [13], [17].

The studies described above have provided a valuable comparison of growth conditions, but these individually-gathered datasets have not been fully integrated to provide a systems biology view of metabolism, which is crucial to understanding methylotrophic growth and predicting metabolic manipulations for industrial benefit. In addition, the dynamics of the response during the transition between growth substrates has not been studied at a global level, and that approach has great potential to provide insights into how the metabolic network is reset to allow methylotrophic growth as well as to uncover general cellular strategies for adaption to environmental change and metabolic stress.

In this study, we address how metabolism responds and adapts under perturbation as a system of interconnected metabolic pathways by adding methanol to a succinate-limited chemostat culture and following the transition of the cells from succinate growth to methanol growth, obtaining data at multiple time points for different layers in the metabolic hierarchy: transcription, enzyme activity, metabolites and fluxes. This integrated analysis provides information about the transient imbalance and the response to carbon starvation the cells experience when they are shifted out of the multi-carbon metabolic mode, the flux redistribution that occurs while the cells are adapting to the new C_1_ metabolic mode, and the relationships between transcript, enzyme activity, metabolite and flux during this transition. In addition, we show that unlike in other organisms, [22]–[23], *M. extorquens* AM1 induces its metabolic pathways in a reverse order (biomass cycles first before the pathways that generate the biomass precursor metabolites), which may be a cellular strategy to prevent the buildup of downstream toxic metabolites. Finally, we show that for those genes/enzymes common to both modes of growth, expression and activity change greatly during the transition, yet metabolite concentrations remain relatively constant underscoring the concept of metabolic setpoints [24].

## Results

### Parameters of the Transition Experiment and Growth Response

**Figure 1:**
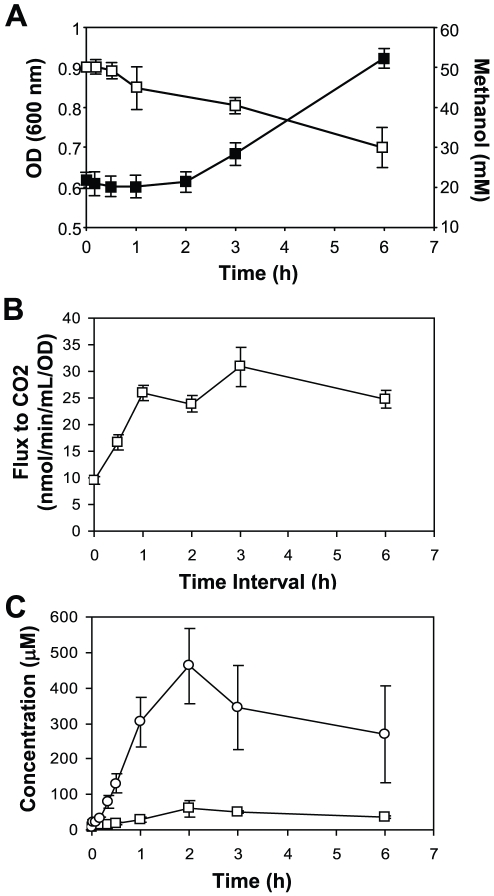
Optical density (OD), rates of carbon flux to CO_2_ and external metabolite measurements before and during the transition from succinate to methanol growth. (A) Change of optical density (filled squares) and methanol concentrations (open squares). (B) Carbon flux to CO_2_ measured using ^14^C-methanol. (C) Formaldehyde (squares) and formate (circles) concentrations in the culture supernatant. Standard deviations are shown as error bars.

Chemostat cultures were chosen as the starting point for the substrate transition experiments due to the ability to maintain reproducible initial growth conditions. Succinate-limited chemostat cultures of *M. extorquens* AM1 were grown to steady state (OD ∼0.63), samples were taken as a zero time point, and then 50 mM methanol was added. Cells were harvested at multiple time points after the transition, up to 6 h, and cellular response was assessed via cell growth, carbon flux measurements, selected enzymatic activity assays, global gene expression profiling and measurement of a suite of targeted intracellular and excreted metabolites. Upon cessation of succinate addition to the growth medium and the subsequent addition of methanol, it can be estimated that the carbon flux to biomass dropped from ∼30 nmol C min^−1^ ml of culture^−1^ (calculated from the dilution rate, see Methods) to ∼0.2 nmol C min^−1^ ml of culture^−1^, which is the measured flux of methanol and CO_2_ carbon to biomass in succinate-grown cells exposed to methanol in batch culture [20]. This drop in flux of over two orders of magnitude would be expected to plunge the culture into carbon starvation initially, until the induction of the methylotrophic assimilatory pathways allowed increased flux to biomass from those routes. In keeping with this expectation, the culture underwent a 2 h lag phase, demonstrated by the relatively unchanged optical density (OD_600_) (Figure 1A), then transitioned to log phase indicating that the culture had started to actively grow on methanol. The methanol concentration in the medium began to drop between 15–30 min post methanol addition, with a concentration of ∼30 mM left at 6 h post transition. Note that since the culture was initially at steady-state under succinate limitation, this protocol effected a transition from succinate to methanol growth without the need to wash the starting culture.

### Global Gene Expression Response to Carbon Source Switch

**Figure 2:**
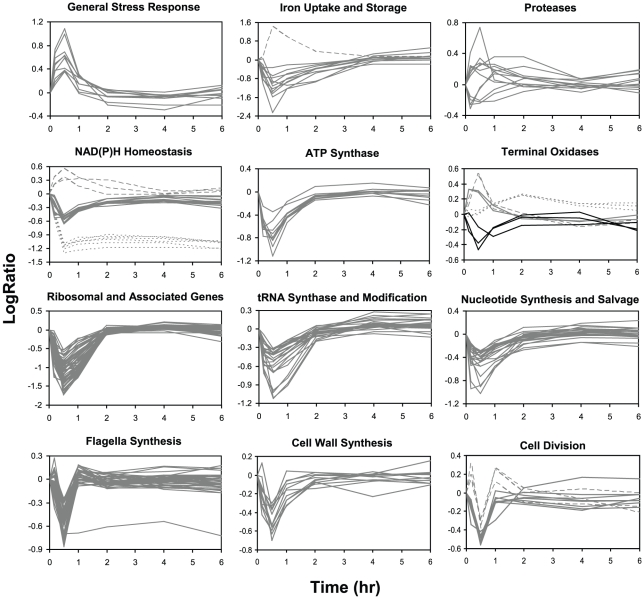
Changes in gene expression of general cellular functions that significantly changed during the transition from succinate to methanol growth. Graphs include genes predicted to be involved in general stress response (top left), iron uptake (top center, solid lines) and storage (top center, dashed line), peptide cleavage (top right), NAD(P)H homeostasis (upper-middle left) including NADH:ubiquinone oxidoreductase (*nuo*, solid lines), transhydrogenase (*pnt*, short dashed lines) and three additional genes (long dashed lines) predicted to encode NADH dehydrogenase 2, NADH:flavin oxidoreductase/NADH oxidase and NAD(P)H quinone oxidoreductase, ATP synthase function (upper-middle center), terminal oxidase function (upper-middle right) including cytochrome c oxidase (*coxABC*, solid black lines), cytochrome bd ubiquinol oxidase (*qxtAB*, solid gray lines), cytochrome d terminal oxidase (*cydAB*, long dashed lines) and cytochrome o ubiquinol oxidase (*cyoABC*, short dashed lines), ribosome function (lower-middle left), tRNA synthesis and modification (lower-middle center), nucleotide synthesis and salvage (lower-middle right), flagella synthesis (bottom left), cell wall synthesis (bottom middle) and cell division (bottom right). LogRatios, intensities, fold changes and p-values are shown for each gene in Table S1.

To gain insights into the global cellular response upon carbon source switch, gene expression data were analyzed to determine the cellular processes (outside of central metabolism) that showed the largest changes in their initial response to methanol addition (Figure 2) and were compared to a set of carbon-starvation response arrays as controls (Table S1). Several classes of genes responded similarly in both conditions, in keeping with the prediction that the cells would experience carbon starvation at the onset of the transition. These included genes whose expression increased in both cases, including predicted stress response genes, predicted genes involved in NADH homeostasis, cytochrome bd ubiquinol oxidase genes, and a predicted bacterioferritin (iron storage) gene. In addition, many classes of genes showed a transient decrease in expression in both conditions, either within the first 30 min or after a transient increase, and then a recovery. This pattern included genes predicted to be involved in synthesis of proteins, fatty acids, lipopolysaccharides, ATP, flagella, cell walls and nucleotide synthesis and salvage, along with cytochrome c oxidase genes and several genes predicted for cell division, chemotaxis, glycosylation, and protease activity. A similar pattern of decrease and recovery was also observed for several methylotrophy genes, discussed below. Genes encoding transhydrogenase, which interconverts NADH and NADPH [25], were significantly down-regulated (15–20 fold) in the methanol addition condition but not in the starvation control, and remain repressed during the time period of methylotrophic growth (Figure 2), consistent with previous steady state studies [14]. Cytochrome d terminal oxidase genes increased independently of the carbon starvation control while the cyotchrome o ubiquinol oxidase genes increased in opposition to the carbon starvation control, where expression decreased ∼5 fold.

### Overview of Central Metabolic Carbon Flow

**Figure 3:**
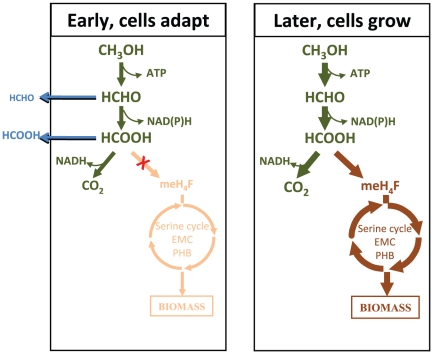
Overview of flux changes through methylotrophic central metabolism during the transition from succinate to methanol growth. Dissimilatory steps are shown in green, assimilatory steps in brown. Methylene-H_4_F is abbreviated as meH_4_F.

In *M. extorquens* AM1, methanol is first oxidized to the toxic metabolite, formaldehyde, then to formate. Formate serves as a branch point and can either be oxidized to CO_2_ producing reducing power, or be converted to methylene-H_4_F, which enters the central metabolic carbon assimilation pathways including the serine cycle, the EMC pathway, the PHB cycle and a portion of the TCA cycle, where essential intermediates are produced for cell growth [1]–[2], [21] (Figure 3). These pathways were analyzed in detail along with two core pathways involved in multi-carbon growth, the TCA cycle and the pentose-phosphate pathway. After methanol was added to succinate-grown cultures, distribution of carbon flow was assessed using 4 methods: growth curve analysis, measurement of flux to CO_2_ using ^14^C-labeled methanol, measurement of methanol, formaldehyde and formate in the culture supernatant and measurement of assimilatory pathway metabolites (described below). Growth did not occur until between 1–2 h post transition (Figure 1A), yet flux of methanol to CO_2_ increased significantly within the first hour, prior to growth (Figure 1B). Formaldehyde and formate concentrations in the supernatant increased until about 2 h, when levels began to decrease (Figure 1C), suggesting that before active growth occurred, both compounds were excreted and then as the cells began to divide, excretion decreased. For the first hour, approximately 1/3 of the carbon from methanol oxidation was in these two pools, the remainder in CO_2_. Note that the peak concentrations, 50 and 450 µM, respectively, are not toxic for *M. extorquens* AM1 [26]–[28]. By 2 h, the total flux to formaldehyde, formate, and CO_2_ of the culture (∼32 nmol min^−1^ [ml at 1 OD]^−1^) was about ¾ of the full flux measured in methanol-grown cells (41.5 nmol min^−1^ [ml at 1 OD]^−1^; [21]). These data suggest that the lag in growth was due to a block in formate assimilation, not production (summarized in Figure 3).

### Visualization of Hierarchical Changes for Central Metabolism

**Figure 4:**
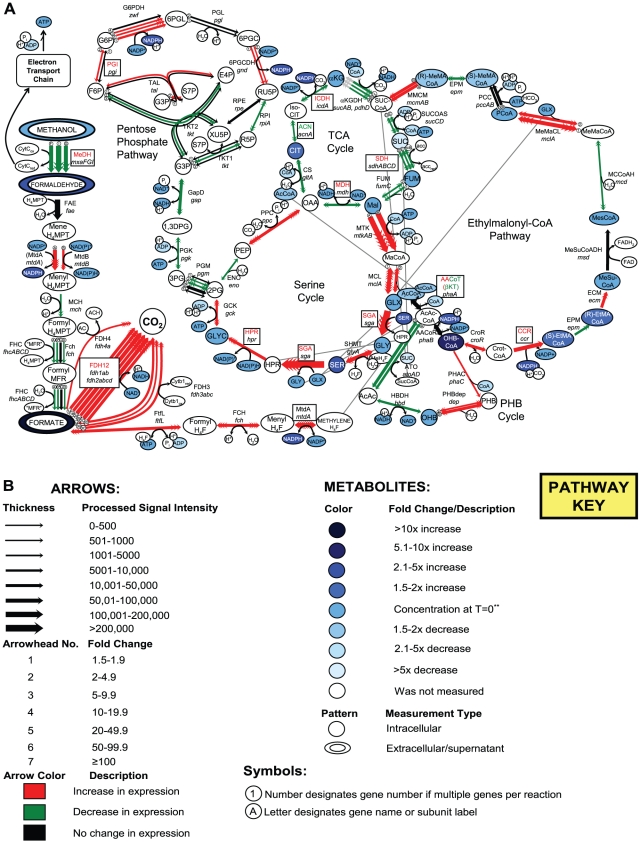
Pathway schematic depicting changes that occurred in measured metabolites, gene expression, and enzymatic activities for central metabolism during the transition from succinate to methanol growth. A boxed gene/protein name indicates the activity of this enzyme was measured. Red lettering for the protein designation indicates an increase in activity; green, decrease; black, no change. Metabolites appearing more than once are connected by gray lines. Changes are shown for (A) the initial response, time  = 10–30 min with (B) serving as a legend. Graphs depicting changes for pre-methanol addition, time  = 0 min; just prior to/at the start of cell growth, time  = 1–2 h; and exponential cell growth, time  = 3–6 h are included in Figure S1. Reaction descriptions are included in Table S2 along with gene expression intensities, LogRatios, fold changes and p-values. Mesaconyl-CoA, ethylmalonyl-CoA, methylsuccinyl-CoA were measured as free acids. **Color at T = 0 represents the concentration before methanol was added for all metabolites except for methanol which was calculated as 50 mM for the initial T = 0 value.

With the introduction of global “omics” level tools, it has recently become possible to investigate multiple layers of an organism's metabolic network during a condition of study. However, the power of these tools can also be a detriment, generating large amounts of data that can often be difficult to integrate and understand as a whole. To facilitate insights and infer meaning regarding the multi-leveled changes and adaptations that the metabolic network of *M. extorquens* AM1 undergoes during the transition from succinate- to methanol-growth, diagrams were constructed that visually compile and summarize each level of data obtained in relation to central metabolism. The initial response to methanol addition (time  = 10–30 min) is shown in Figure 4A with 4B serving as a legend. Diagrams depicting the metabolic state prior to methanol addition (time = 0 min), response just prior to/at the start of cell growth (1–2 h) and during log phase cell growth (3–6 h) are included in Figure S1. These diagrams depict information about gene expression intensities (arrow thickness) and fold changes (number of arrow heads), changes in measured metabolite concentrations (color shadings), and enzymatic activities (color shadings of boxed protein names), providing insight into both the metabolic changes themselves and the level at which those changes occurred. While only semi-quantitative due to possible differing labeling and hybridization efficiencies that could occur during microarray experiments, information on gene intensities is provided since the intensity data aid in pathway interpretation of possible carbon flow. Analysis of fold changes alone can be misleading if for example, no change in expression occurs for a gene, yet expression of that gene is high, or if a gene has a significant change in expression but expression is extremely low.

### Response of Measured Nucleotide Pools

**Figure 5:**
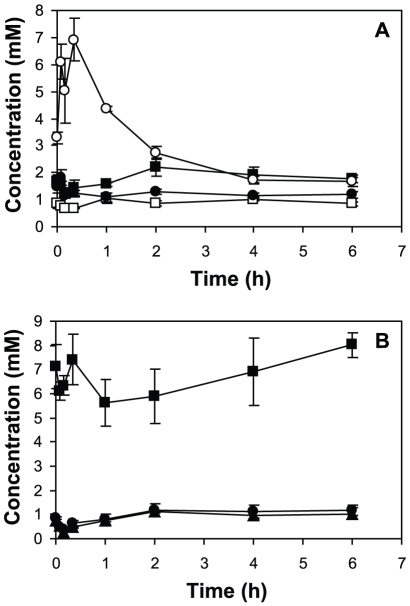
Changes in nucleotide concentrations before and during the transition from succinate to methanol growth. (A) Changes of pyridine nucleotides: NAD (filled squares), NADH (open squares), NADP (filled circles), NADPH (open circles), (B) changes of adenine nucleotides: ATP (filled squares), ADP, (filled circles), AMP (filled triangles). Standard deviations are shown as error bars.

The adenine and pyridine nucleotide pools reflect the metabolic state of the cell. As expected for a growth downshift experiment, these compounds all showed significant changes, with most values changing by 50–100% during the time course (Figure 5). Consistent with the prediction that methanol growth is reducing power limited and succinate growth is energy limited [12]–[13], NADPH increased immediately, in keeping with an initial major downshift in biomass synthesis, peaked at the 30 min timepoint, then decreased by 6 h to a lower value than the initial value. ATP decreased immediately, in keeping with the downshift in carbon flow through the TCA cycle, then increased, then decreased, and after 1 h, slowly increased to a final value greater than that for succinate growth. The other nucleotides all decreased immediately, then rose and either stayed constant or decreased slightly, a pattern reminiscent of the starvation-induced gene expression data.

### System Response for Oxidation of Methanol to Formaldehyde

**Figure 6:**
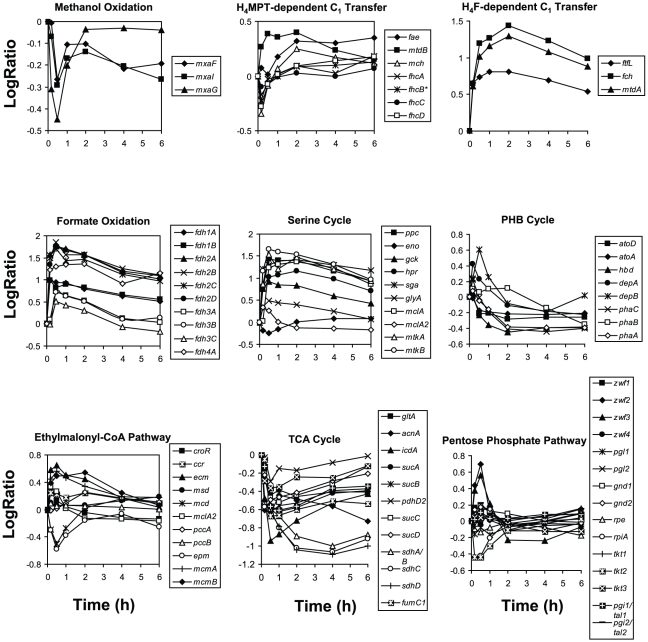
Changes in gene expression for central metabolic pathways during the transition from succinate to methanol growth. Graph include genes encoding methanol dehydrogenase (*mxaFI*) and cytochrome *c*_L_ (*mxaG*) (top left), H_4_MPT-dependent (top center) and H_4_F-dependent (top left) C_1_ transfer genes, formate oxidation genes (middle left), serine cycle genes (middle center), PHB cycle genes (middle left), EMC pathway genes (bottom left), TCA cycle genes (bottom center) and pentose phosphate pathway genes (bottom right) after addition of methanol (50 mM, final concentration) to succinate-limited chemostat-grown cells.

**Figure 7:**
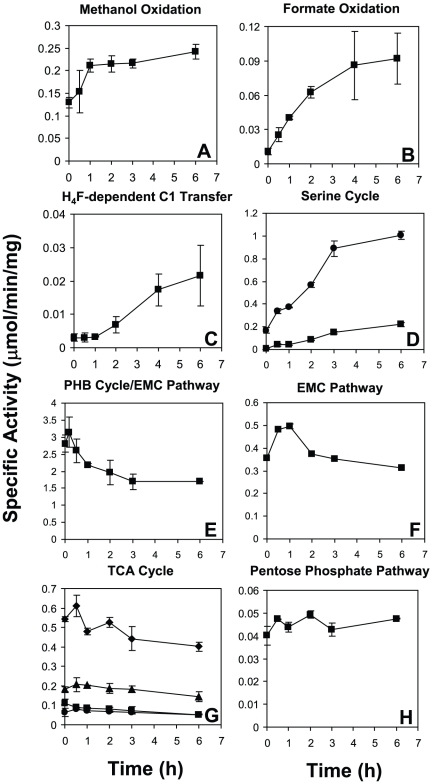
Changes in enzymatic activity before and during the transition from succinate to methanol growth. Measured activities include (A) methanol dehydrogenase activity, (B) NAD-dependent formate dehydrogenase activity (Fdh1,2) (C) methylene-H_4_F dehydrogenase activity (MtdA), (D) serine-glyoxylate aminotransferase (Sga, squares) and hydroxypyruvate reductase (Hpr, circles), (E) β-ketothiolase (βKT, encoded by *phaA*), (F) crotonyl-CoA reductase (Ccr), (G) malate dehydrogenase (Mdh, diamonds), isocitrate dehyhdrogenase (Icd, triangles), aconitase (Acn, squares) and succinate dehydrogenase (Sdh, circles). Standard deviations are shown as error bars.

Methanol is oxidized to formaldehyde by methanol dehydrogenase. In the succinate-limited cultures, the transcripts for the genes encoding the methanol dehydrogenase subunits (*mxaF*, *mxaI*) and its cytochrome (*mxaG*) were very high (Figure S1A, expression intensity values are given in Table S2). Upon methanol addition, these transcripts initially decreased 2–3 fold (Figure 6), then increased and had recovered to near starting levels by 2 h, when the cells began to grow, a pattern similar to the carbon starvation response noted earlier. The other genes involved in generating the active form of methanol dehydrogenase (*mxaACDEHJKLRS*) showed a similar pattern (see Table S2). However, enzymatic activity of methanol dehydrogenase did not mirror transcriptional changes. Activity in the succinate-grown cells was 129 nmol min^−1^ mg protein^−1^ initially and increased over the 6 h of the experiment after methanol was added, to 242 nmol min^−1^ mg protein^−1^ (Figure 7A).

### System Response for Oxidation of Formaldehyde to Formate

To oxidize formaldehyde, *M. extorquens* AM1 uses the cofactor, tetrahydromethanopterin (H_4_MPT) as a carrier of one-carbon units, converting formaldehyde to formate in 5 enzymatic steps as shown in Figure 4A. Due to the unavailability of H_4_MPT and its conjugates, enzyme activities in this pathway were not measured. The transcript of the first enzyme required for the oxidation of formaldehyde (Fae, formaldehyde activating enzyme) was high in the succinate grown cells (Figure S1A, Table S2) and increased 2.2-fold over 6 h (Figure 6). This enzyme is key to preventing formaldehyde from building up to toxic levels inside the cell [29]. The next step in the pathway involves MtdB, which generates NAD(P)H. In addition, a second enzyme with activity towards H_4_MPT and NADP, MtdA, is present in *M. extorquens* AM1, but the main role of this enzyme appears to be in the H_4_F-dependent C_1_ transfer pathway [21], [30], so it will be discussed below, in that section. The gene encoding MtdB increased in expression immediately, leveled out at 30 min, and then decreased slowly over the rest of the time course. However, expression of the remaining genes of the formaldehyde oxidation pathway (H_4_MPT-dependent C_1_ transfer pathway) decreased initially (*mch, fhcABCD*, Figure 6) similar to the genes for methanol dehydrogenase, and similar to the carbon starvation response. In general, expression of these genes returned to starting levels between 1–2 hours, increasing slightly or remaining constant through the remaining 6 hrs.

### System Response for Oxidation of Formate to CO_2_

Oxidation of formate to CO_2_ is carried out by formate dehydrogenase (Fdh). *M. extorquens* AM1 contains 4 known Fdhs, and transcripts for the genes encoding those Fdhs increased immediately and considerably, peaking in expression levels between 10 and 30 min post transition, then decreased over time (Figure 6). It has not yet been possible to assay the activities of Fdh3 and Fdh4 [26]–[27], but the combined activities of the NAD-dependent enzymes, Fdh1 and Fdh2, increased immediately and continued to increase over the 6 h time period (Figure 7B), correlating with the increase in transcripts and the flux to CO_2_.

### Response of the H_4_F-dependent C_1_ Transfer Pathway for Conversion of Formate to Methylene-H_4_F

Three enzymes are required to convert formate to methylene-H_4_F: formyl-H_4_F ligase (FtfL), methenyl-H_4_F cyclohydrolase (Fch) and methylene-H_4_F dehydrogenase (MtdA). Transcripts for each of these genes responded in a similar manner, increasing immediately until 2 h, when the cells began to grow (Figure 6). Then the transcripts decreased but remained high over the course of the experiment. MtdA activity was assayed and showed a 1 h lag in activity (Figure 7C), though expression of this gene increased immediately and rose to levels 20-fold above the starting point.

### Response of the Carbon Assimilation Pathways

The serine cycle and EMC pathway are the main pathways by which *M. extorquens* AM1 incorporates C_1_ compounds from methanol into biomass. The EMC pathway overlaps with the PHB, Serine and TCA cycles (Figure 4A).

#### Serine cycle

**Figure 8:**
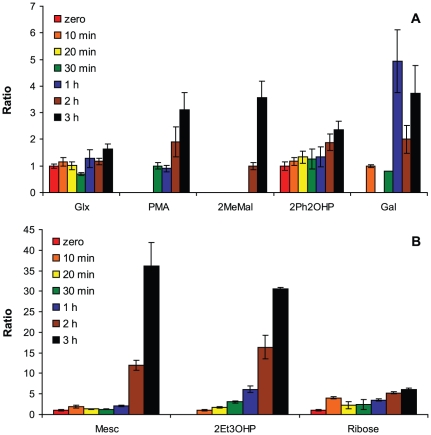
Metabolite ratios for selected metabolites for which concentrations are not available measured before and during the transition from succinate to methanol growth. Ratios for metabolites that changed up to 5-fold are graphed in (A) and those that increased >5-fold are depicted in (B). Ratios are set to 1 for initial detection. Abbreviations are as follows: Glx, glyoxylate; PMA, propylmalonate; 2-MeMal, 2-methylmalate; 2-Ph2OHP, 2-phenyl-2-hydroxypropionate; Gal, galactose; Mesc, mesaconic acid; 2Et3OHP, 2-ethyl-3-hydroxypropionate. Standard deviations are shown as error bars.

With the exception of enolase, expression of the serine cycle genes increased immediately and peaked at 10 min post switch (Figure 6). As is well-known [31]–[32], hydroxypyruvate reductase (Hpr) and serine-glyoxylate aminotransferase (Sga) had low activities in cells growing on succinate compared to methanol. After methanol addition, their activities increased considerably (6-fold and 44-fold, respectively) and continuously over the next 6 hours (Figure 7D), as previously reported for batch culture experiments [33]. Expression of the enolase gene decreased initially and then increased back to its original pre-transition level (Figure 6), similar to the carbon starvation response. While expression of enolase stands apart from the other serine cycle genes, unlike the other serine cycle genes, it is essential for growth on both succinate and methanol [10]. With the exception of serine, which responded in a manner similar to other amino acids (alanine, threonine, aspartate and phenylalanine; Table 1), measured serine cycle intermediates (glycerate, malate, glyoxylate and glycine) did not significantly change between the time of methanol addition and cell growth (Table 1, Figure 8). Most of the amino acids measured showed an increase within the first 30 min, then decreased to their approximate initial concentrations, which correlated with an initial increase in transcription of a number of putative protease genes (Figure 2), suggesting this response may have been due to protein turnover early in the transition.

**Table 1:** Intracellular concentrations of target metabolites from cells during transition from succinate growth to methanol growth.

		zero	10 min	20 min	30 min	1 h	2 h	3 h
Pathway	Metabolite			Concentration (mM)				
TCA	Suc	12.2±0.7	3.7±2.5	2.1±0.7	2.9±0.8	5.0±0.3	2.2±0.3	2.3±0.9
	Fum	0.5±0.1	0.6±0.1	0.5±0.0	0.5±0.1	0.5±0.1	0.5±0.1	0.5±0.1
	Mal	1.6±0.4	1.7±0.4	1.2±0.2	1.3±0.4	1.0±0.4	0.9±0.1	1.2±0.4
	2KG	1.4±0.3	1.5±0.2	1.7±0.3	1.4±0.4	1.5±0.3	2.3±0.3	2.5±0.4
	Cit	1.8±0.5	2.0±0.4	3.3±0.4	1.8±0.4	2.2±0.6	2.1±0.4	2.4±0.5
	Pyr	3.7±0.4	4.4±1.2	3.9±0.3	4.6±0.5	5.1±0.7	4.8±0.4	4.9±0.3
Serine	Glyc	1.7±0.3	2.5±0.6	2.0±0.5	2.1±0.3	2.0±0.4	1.8±0.2	2.2±0.7
	Ser	4.0±1.5	4.5±1.1	4.2±1.0	16.7±10.4	10.1±6.8	3.3±0.8	2.9±1.7
	Gly	0.8±0.1	0.8±0.1	0.8±0.1	0.9±0.2	1.0±0.1	1.0±0.0	1.2±0.1
PHB	OHB	3.0±0.2	3.3±0.4	3.4±0.2	3.3±0.5	4.4±0.7	4.0±0.5	4.3±0.9
	OHB-CoA	0.1±0.1	0.2±0.1	NM^a^	0.4±0.1	0.3±0.1	NM	0.3±0.1
EMC	EtMA	0.6±0.1	0.8±0.1	1.1±0.1	1.0±0.1	1.2±0.1	2.1±0.2	3.1±0.4
	MeSuc	0.6±0.0	0.7±0.1	0.8±0.0	0.7±0.1	0.8±0.0	1.7±0.1	4.3±0.8
	P-CoA	0.1±0.1	0.1±0.1	NM^a^	0.1±0.1	0.2±0.1	NM	0.3±0.1
	MeMA	0.3±0.0	0.1±0.1	NM	0.1±0.1	0.2±0.1	NM	0.3±0.1
Other^b^	Ac-CoA	0.3±0.1	0.2±0.1	NM	0.2±0.1	0.2±0.1	NM	0.3±0.1
	M-CoA	0.1±0.1	0.3±0.1	NM	0.1±0.1	0.2±0.1	NM	0.2±0.1
	CoA	2.6±0.2	0.7±0.5	NM	1.5±0.2	1.2±0.3	NM	1.4±0.5
	Ala	7.6±0.7	9.4±2.0	9.4±1.1	14.0±1.5	12.9±4.1	9.7±0.3	10.0±0.6
	Val	1.0±0.1	0.7±0.2	0.9±0.1	1.4±0.4	1.5±0.6	1.2±0.1	1.3±0.2
	Leu	0.6±0.0	0.6±0.2	0.8±0.0	0.9±0.1	0.9±0.4	1.1±0.1	1.4±0.3
	Ile	0.4±0.1	0.5±0.1	0.6±0.0	0.7±0.1	0.7±0.1	0.5±0.0	0.6±0.0
	Pro	1.3±0.2	1.3±0.3	1.6±0.1	1.6±0.3	1.4±0.4	1.1±0.2	1.0±0.4
	Thr	3.1±0.4	3.4±0.6	3.4±0.6	6.3±3.4	5.2±1.1	3.0±0.5	3.3±0.7
	Asp	4.9±1.5	4.9±1.3	4.9±0.5	5.9±1.1	6.2±3.1	2.2±2.0	2.3±2.4
	Phe	0.3±0.0	0.4±0.1	0.7±0.1	0.5±0.2	0.4±0.2	0.2±0.1	0.2±0.1

Numbers are averages of nine replicates, and standard deviations are listed as ± sd. The abbreviations used in the table are defined as follows: Suc = succinate; Fum = fumarate; Mal = malate; 2KG = 2-ketoglutarate; Cit = citrate; Pyr = pyruvate; Glyc = glycerate; OHB = 3-hydroxybutyrate; OHB-CoA = 3-hydroxybutyryl-CoA; EtMA = ethylmalonate; MeSuc = methylsuccinate; P-CoA = propionyl-CoA; MeMA = methylmalonate; Ac-CoA = acetyl-CoA; M-CoA = malonyl-CoA.

a NM  =  not measured.

b Other refers to measured metabolites that are not depicted in the central metabolic diagrams (Figure 4) or are components of multiple central metabolic pathways.

#### EMC pathway

The majority of the EMC pathway genes were immediately and highly induced, peaking at 10–30 min and then returning to just above pre-transition levels (Figure 6). In contrast, metabolites of the EMC pathway (measured as their non-CoA derivatives) hydroxybutyrate, ethylmalonate, methylsuccinate, mesaconate, 2-methylmalate, did not show significant increases until the 2 or 3 h timepoint, while methylmalonyl-CoA and propionyl-CoA did not show significant increases until the 1 or 2 h timepoint (Table 1, Figure 8). Expression of *mcd* and *epm* encoding, respectively, mesaconyl-CoA hydratase and epimerase, mirrored each other and did not follow the pattern of the other EMC genes, decreasing immediately and returning to starting levels or higher by 4 h post transition (Figure 6), reflecting the carbon starvation response genes. Comparisons of expression changes of *mcd* and *epm* in steady-state succinate and methanol grown cells were consistent with these data, being similar in both growth conditions [14]. Between 1–2 h, carbon flux occurred via the EMC pathway, evidenced by the accumulation of mesaconate, methylsuccinate, ethylmalonate, methylmalonate, methylmalonyl-CoA and propionyl-CoA (Figure 8, Table 1, summarized in Figure S1C), and cell growth occurred. Expression of *msd*, encoding methylsuccinyl-CoA dehydrogenase, which is known to be required for growth on C_1_ compounds [34], did not significantly change, yet expression intensities suggested that this gene might be highly expressed under conditions of both methanol and succinate growth. With few exceptions, both array data sets (biological replicates) were in agreement regarding range of intensity and fold change. Notable exceptions were *ccr* and *croR*, encoding respectively, crotonyl-CoA reductase and crotonase, which increased by a small amount (1.8-fold for both) in the first array data set but not the second.

The activities of two enzymes of the EMC pathway, β-ketothiolase (βKT; encoded by *phaA*) and crotonyl-CoA reductase (Ccr; encoded by *ccr*) were measured. βKT activity, which overlaps with the EMC and PHB cycles, increased slightly initially then decreased to ∼60% of the original activity (Figure 7E), mirroring expression of *phaA* (Figure 6). 3-hydroxybutyryl-CoA, which is an intermediate of both the EMC pathway and PHB cycle, showed a similar pattern, increasing initially and then remaining relatively constant (Table 1). Ccr activity was high in succinate grown cultures (356 nmol min^−1^ mg protein^−1^, Figure 7F), increased 1.4-fold by 1 h, and then decreased to just below starting levels by 6 h, consistent with the minimal changes in transcripts noted above.

Using Fisher ratio analysis to assess metabolite differences between two samples, a number of metabolites were previously identified (2-methylmalate, propylmalonate, 2-phenyl-2-hydroxy propionate, 2-ethyl-3-hydroxypropionate, ribose and galactose) as being significantly higher in methanol-grown cells than succinate-grown cells [15]. These metabolites responded similarly to the EMC pathway intermediates and are shown in Figure 8, but their role in C_1_ metabolism, if any, is unknown.

#### PHB cycle

Poly-β-hydroxybutyrate (PHB) accumulates in *M. extorquens* AM1 as a carbon and energy storage compound [35]–[36]. The *phaA* and *phaB* genes, which are required for PHB synthesis, are shared with the EMC pathway. Expression of these genes increased slightly initially, then decreased over the course of the experiment with *phaA* decreasing between 30 min–1 h and *phaB* decreasing between 4–6 h (Figure 6). These gene products produce 3-hydroxybutyryl-CoA, which is a precursor to PHB as well as an intermediate of the EMC pathway. While expression of these genes did not change much initially and activity of βKT increased minimally (Figures 6,7E), concentration of 3-hydroxybutyryl-CoA increased 4-fold within the first 30 min after methanol addition (Table 1). Expression of the depolymerase genes (*depA*, *depB*), which hydrolyze PHB, increased 2.6- and 4.1-fold respectively at 10 min and then decreased over the time course. Genes involved in the further degradation of PHB (*hbd*, *ato*) decreased after 10 min post methanol addition and remained lower than initial levels.

### Response of the TCA Cycle

Expression of all TCA cycle genes (*sdhABCD*, *sucAB*, *icdA*, *acnA*, *gltA*, *mdh*, *fumC*) decreased severely (Figure 6) but intensities were still well above background levels throughout the experiment (Table S2). However, aconitase (Acn) was the only measured enzyme that exhibited a significant decrease in enzyme activity (48% decrease) while other enzyme activities increased slightly and then returned to just below their starting activity (Icd, 79%; Sdh, 82%, Mdh; 74% decrease; Figure 7G). In keeping with the enzyme activity data, except for succinate, levels of the measured TCA cycle metabolites remained relatively constant, not showing distinct trends (Table 1). Succinate was high initially at time zero as expected since it was the carbon source, and decreased from 12.2 mM to 3.7 mM within 10 min of stopping medium flow (Table 1).

### Response of the Pentose Phosphate Pathway

Predicted genes for the pentose phosphate pathway (*zwf*, *pgl*, *gnd*, *fbp*, *pgi*, *tkt*, *fba*. *rpe*, *rpi*) exist in *M. extorquens* AM1, often in duplicate or triplicate, yet the operation of this pathway has not been thoroughly studied. Gene expression intensities suggested that enzymes involved in this pathway are present during both succinate and methanol growth, with different portions of the pathway up-regulated during the transition. 2 of the 4 candidate *zwf* (glucose-6-phosphate dehydrogenase) genes were significantly up-regulated after methanol was added (4–5 fold) while 1 of 3 predicted *tkt* (transketolase) genes along with a predicted *rpiA* (ribose-5-phosphate isomerase) gene were down-regulated (∼3 fold) (Figures 4A, 6). Expression of a third putative *zwf* gene along with *gnd1* (6-phosphogluconate dehydrogenase), *gnd2* and *tal2/pgi2* (transaldolase/phosphoglucose isomerase) increased slightly (1.5–1.7 fold) (Figures 4A, 6). Activity of phosphoglucose isomerase (PGI) was measured and increased minimally (Figure 7H). This result was generally consistent with the gene expression studies. Expression of *pgi2* did increase but peaked only at ∼1.7-fold while *pgi1* increased less than 1.3-fold (Figures 4A, 6).

## Discussion

When cells are shifted from one metabolic mode to another, significant remodeling of metabolic networks occurs. With the advent of global profiling capabilities, it is now possible to study metabolic response using a systems biology approach. In this paper, we used whole genome microarrays in conjunction with targeted enzyme assays and metabolite and flux measurements to assess the dynamic response that *M. extorquens* AM1 undergoes when switching from multi-carbon growth to single-carbon growth. Integrating datasets targeting multiple layers in the metabolic hierarchy has generated a much more complete picture of how the cells respond and adapt to changes in their environment than any of the datasets alone, and has provided insights into the mechanisms of this response.

To switch from succinate to methanol growth, energy metabolism shifts from NADH oxidation via NADH dehydrogenase to a cytochrome-based electron transport system that accepts electrons from methanol dehydrogenase during methanol oxidation [1]. Carbon metabolism shifts from the TCA cycle and its associated pathways to a methylene H_4_F- and CO_2_-based assimilatory scheme involving the serine cycle and the EMC pathway [1], [12], [17], while NADH production shifts from the TCA cycle to the methylene H_4_MPT dehydrogenase and formate dehydrogenase enzymes ([1], [10]; Figures 3,4A). To accomplish this shift, over 100 genes must be up-regulated in *M extorquens* AM1, producing over 50 enzymes and a number of regulatory proteins [10], and the cell must accommodate high flux through toxic metabolites, such as formaldehyde, glyoxylate, and glycine.

### Carbon Starvation

Our time course experiments demonstrated that when the cells were transitioned from a state of succinate growth to methanol growth, they underwent a period of transient carbon starvation followed by periods of recovery and growth. During the starvation phase, the cells were thrown out of metabolic balance and growth ceased, and during the recovery period, all of the methylotrophic metabolic pathways rapidly changed to allow the cells to shift metabolic modes.

During the starvation period (0–30 min), gene expression patterns largely reflected those for a starvation control. In both the starvation control and the methanol switch experiment, expression of genes predicted to be involved in many core cellular processes such as cell structure synthesis, ribosome synthesis and protein production, flagellar synthesis, and iron uptake were down-regulated, conserving energy. Stress response genes were up-regulated along with several protease genes, which correlated with the increase in free amino acid pools detected in cell extracts. Expression of cytochrome c oxidase genes decreased by 30 min and then rose by 2 h mirroring expression of methanol dehydrogenase genes and cytochrome c (encoded by *mxaG*) used during methanol metabolism suggesting the possibility of a coordinated response. Immediately after the transition, ATP dropped, suggesting that energy metabolism was curtailed, while NADPH rose transiently, in keeping with a drop in biosynthetic consumption, and then dropped, consistent with a drop in production.

### Transient imbalance

Cells growing on succinate maintain significant capacity to oxidize methanol to formaldehyde and then to formate. For the first h after the switch from succinate to methanol, the cells excreted about 1/3 of the total production of these compounds into the medium, mainly as formate. This result suggests that the capacity to consume formate was initially less than the formate production capacity, but by converting most of the formaldehyde to formate and excreting the excess, the cells could survive the transition without buildup of formaldehyde to toxic levels. It is likely that excretion serves as a buffer for flux capacity to these intermediates, allowing the cell to adjust formate consumption capacity according to need, rather than maintaining a high constitutive capacity. During this time period, the formate dehydrogenases rapidly induced and the flux of methanol to CO_2_ reached nearly full capacity by the time the culture started growing, at about 2 h. By maintaining significant methanol oxidation capacity in the absence of substrate, the cells can take advantage of transient methanol sources to obtain energy, without committing to the considerable effort of inducing the entire system of methylotrophic metabolism.

### Post-transcriptional regulation

After the first 30 min, virtually all of the genes involved in methylotrophy began to induce, as expected. However, a number of examples were identified in which enzyme activities did not reflect transcript changes, suggesting post-transcriptional regulation. These included: 1) methanol dehydrogenase, for which enzyme activity increased rapidly during the time that transcripts dropped, suggesting a mechanism of enzyme activation; 2) methylene H_4_F dehydrogenase, for which transcripts increased 14-fold but enzyme activity stayed constant for the first hour, suggesting a mechanism of post-transcriptional inhibition, either of translation or of enzyme activity, and 3) TCA cycle enzymes (Icd, Sdh, and Mdh), for which the activities stayed relatively constant while transcripts dropped, even after cell division, suggesting either a mechanism of enzyme stabilization or activation.

### Flux redistribution

Surprisingly, during the time period from 30 min to 2 h while the flux capacity from methanol to CO_2_ was increasing, net flux through methylotrophy assimilatory pathways to biomass did not occur, even though both transcripts and enzyme activities increased significantly. Since intermediates of the EMC pathway did not accumulate, the assimilatory block must have occurred before that pathway. A candidate step for this block was identified, the conversion of methenyl H_4_F to methylene H_4_F by methylene H_4_F dehydrogenase, since as noted above, this enzyme did not increase in activity during this time period. Once this activity increased, flux to biomass occurred.

These results suggest a metabolic strategy in which the assimilatory pathways are primed to receive assimilatory carbon, but carbon flux occurs only after they are poised and ready, and only after the dissimilatory flux has increased substantially. Such a strategy might decrease the likelihood of accumulation of intermediates produced in the biomass cycles that could inhibit growth, such as glyoxylate and glycine [8]–[9], ensuring a smooth transition between these two metabolic modes. Our measurements showed that glyoxylate and glycine did not show major changes during this transition.

Another hypothesis generated from these data was that *M. extorquens* AM1 may fine-tune the levels of these toxic metabolites through the Mcd step (mesaconyl-CoA hydratase) in the EMC pathway, which produces/regenerates the glyoxylate required to drive the serine cycle. The expression pattern of *mcd* was notable, since it decreased significantly initially, and only slowly returned to a level slightly above the succinate level. Once intermediates of the EMC pathway began to accumulate, those generated before the Mcd step accumulated to a much higher level than those generated after the Mcd step, consistent with a flux bottleneck at this step. Because glyoxylate is toxic at high levels, it would be advantageous for the cell to regulate the production and consumption of this metabolite. Since glyoxylate is the precursor to glycine during methylotrophic growth, by controlling glyoxylate levels, both of these potentially toxic metabolites are managed. This potential flux bottleneck at the Mcd step would allow the cells to control the balance between need and toxicity for glyoxylate and is an interesting candidate for further study.

Another possible metabolic strategy for carbon partitioning during this transition suggested by these results involved 3-hydroxybutyryl-CoA. This metabolite is an intermediate for both PHB synthesis (carbon storage) and the EMC pathway (carbon assimilation), and is thus a key branchpoint. Succinate-grown cells contain higher levels of PHB than methanol-grown cells [13], and so during the transition, a shift must occur that redirects flux from PHB synthesis to the EMC pathway. Our results showed that 3-hydroxybutyryl-CoA increased almost 6-fold in the first 30 min, before significant increases were observed for the EMC pathway intermediates, at the time when PHB synthesis gene expression was beginning to decrease. In other organisms, the partitioning of acetyl-CoA flux between PHB synthesis and assimilation is controlled by levels of NADPH or the ratio of NADPH/NADP [37]–[38]. The accumulation of 3-hydroxybutyryl-CoA correlated with the increase in NADPH levels and the NADPH/NADP ratio seen during the first 30 min after methanol addition, suggesting that this mechanism of control may also be present in *M. extorquens* AM1. Later, when flux began to increase through the EMC pathway, both 3-hydroxybutyryl-CoA and NADPH levels dropped. By decreasing flux into the PHB cycle first, then increasing the enzymes of the EMC pathway, the cells could effectively shunt 3-hydroxybutyryl-CoA into primary assimilation and away from synthesis of the storage product, PHB.

### Modular response

The results presented here showed that in general, each pathway responded as a separate module, with gene expression changes and enzymatic activities responding as a group. One notable exception was the EMC pathway, in which the majority of the genes increased during the transition yet the patterns of this increase differed (Figure 6).

Another example was the H_4_MPT-dependent C_1_ transfer pathway in which expression of *mtdB* and *fae* stood out as different from the other genes involved in the oxidation of formaldehyde to formate. Fae is present in the cell at very high levels [18]–[19] and likely is involved in keeping intracellular levels of free formaldehyde low. The rapid increase of *fae* transcription may reflect the need to respond to a sudden increase in flux from methanol to formaldehyde. Likewise, MtdB is the first NAD(P)H-generating enzyme in the oxidative pathway, and it responded more similarly to the other NADH-generating step, the formate oxidation pathway. It may be advantageous to the cell to control reduced pyridine nucleotide-generating capacity separately from the other oxidative steps, and act to increase that capacity immediately upon downshift.

### Metabolic set points

These datasets also revealed another major feature, which is the prevalence of metabolic set points. During the transition from succinate to methanol growth, central metabolic pathways change significantly, with correspondingly large changes in flux through these pathways, on the order of 10-fold [20]. Such changes might be expected to reflect transient swings in metabolite concentrations as the enzyme requirements and activities change. However, the concentration of those metabolites that are involved in both succinate and methanol growth such as fumarate, malate, α-ketoglutarate, citrate, pyruvate and glycerate stayed remarkably constant, with less than 2-fold changes during the transition. A similar phenomenon has been reported for *E. coli* wild-type and mutant strains in steady-state cultures under different growth conditions, and these authors suggested that metabolic setpoints may be a mechanism to maintain metabolic robustness in a changing environment [24]. Metabolites that were required for growth on both succinate and methanol but did not appear as metabolic setpoints in this study include succinate and serine. Succinate decreased from 12.2 mM to 3.7 mM within 10 min of stopping medium flow, consistent with the termination of succinate as the carbon source. Serine responded similarly to other amino acids which correlated with an increase in protease gene expression (Table 1, Figure 2).

### Summary

In summary, the multi-level datasets we have generated in this study coupled to our pathway-level analysis have resulted in new insights into the metabolic strategies carried out to effect this shift between two dramatically different modes of growth, one energy-limited (succinate), and the other reducing-power limited (methanol). Analysis of the dynamic response of modular sections of the central metabolic network is an important approach to determine how cells respond and adapt to carbon substrate perturbation. Our work showed that the cells use a “down-stream priming” strategy, inducing biomass cycles before formate production to carry out this transition in a way that does not accumulate toxic intermediates to inhibitory levels. In addition, this work has uncovered key candidates for regulatory control points, as a further step towards understanding metabolic adaptation and the cellular strategies employed to maintain metabolic setpoints.

## Materials and Methods

### Materials

All chemicals, enzymes and chemical standards were obtained from Sigma-Aldrich (St. Louis, MO, USA) unless otherwise specified. Analytical- reagent grade potassium dihydrogen phosphate and sodium dihydrogen phosphate were obtained from Fisher Scientific (Hampton, NH). Absolute ethanol and HPLC-grade methanol (Sigma, St. Louis, MO, USA) were used for extraction procedures and preparation of buffer for HPLC analysis. Nanopure (Barnstead Life Sciences model, Thermo Fisher Scientific, Waltham, MA) water was used in the preparation of media, buffer, standard and sample solutions. To prepare formaldehyde, 0.15 gm of paraformaldehyde was resuspended in 10 ml nanopure water in a glass vial, which was then stoppered, crimp sealed and autoclaved for 5 min.

### Chemostat cultivation

For a starting point (time  = 0 in), the carbon-shift experiment utilized cells from a steady-state chemostat culture of *M. extorquens* AM1 growing on limiting succinate. *M. extorquens* AM1 was cultivated in a minimal medium [14] containing 3.7 mM succinate as a growth-limiting nutrient in a 2.2-liter bench-top BioFlo 110 Modular Fermentor (New Brunswick Scientific) with a 1 liter working volume, resulting in an OD_600_ of ∼0.63. Dilution rate was maintained at 0.163 h^−1^. Other parameters of the chemostat operation were the same as described previously [13]. After the culture reached steady-state, the cells were manipulated in two ways: for enzyme assays, flux measurements and nucleotide determinations, the flows were stopped and methanol was added to the succinate-limited steady-state culture to a final concentration of 50 mM. For RNA isolation, steady-state cells were transferred to flasks before 50 mM methanol addition and incubated at 28°C. For each approach, cells were harvested at different time points as described in the text.

### Transcriptomics

Expression studies were carried out using 60-mer oligonucleotide custom designed Agilent arrays (Santa Clara, CA) as previously described [14]. RNA was extracted immediately and digested with DNase I as described previously [14]. cDNA production and labeling, hybridization and array scanning were carried out by MOgene (St. Louis, MO), using the DNase I-digested RNA. Data extraction and initial processing were as previously described [14]. For the first biological replicate, cells were transferred to a flask, methanol was added (50 mM) and time points were harvested at 0, 10, 30, 60, 120, 240 and 360 min post methanol addition, and each time point included a technical duplicate (dye-swap). Most genes in this study peaked in their induction or repression by 2 h. To confirm the array data expression trends and peak times, a second biological replicate (second chemostat culture) was repeated for the 10, 30, 60 and 120 min time points, each also with a technical duplicate (dye-swap). For carbon starvation arrays, cells were transferred to a flask with no additional carbon and incubated for 30 min prior to harvest. For convenience, time point RNA samples were compared against RNA prepared prior to the transition, directly harvested from the chemostat (time zero, not transferred to flask). LogRatios were adjusted using a time zero flask transfer comparison to remove affects from flask transfer thus setting the time zero expression ratio at 1. Both raw and adjusted data are included in Tables S1, S2. Intensity and LogRatio data were used to determine peak intensities and fold changes to generate the metabolic diagrams. A p-value cutoff of 0.001 (Log p-value ≤−3) was used for validity of expression fold changes. Raw and processed gene expression data are available at the NCBI Gene Expression Omnibus and are accessible through GEO Series accession number GSE22031.

### Measurement of methanol in culture supernatant

Methanol concentration in the growth medium was measured according to Mangos and Haas [39] with a minimum of 3 replicates. The method used alcohol oxidase (EC 1.1.3.13), peroxidase (EC 1.11.1.7) and 2,2′-azinobis-(3-ethylbenzthiazoline-6- sulfonic acid) (ABTS) (Calbiochem, EMD Biosciences, Gibbstown, NJ) to determine methanol concentrations.

### Measurement of formaldehyde and formate in culture supernatant

#### Formate measurements

Formate was measured as previously described for 3 biological replicates [27].

#### Formaldehyde measurements

Formaldehyde was measured for 3 biological replicates using the Nash method [40] in which formaldehyde concentration is proportional to the absorbance at 410 nm.

### Enzyme assays

Cells for enzyme activities (50 ml) were harvested by centrifugation at 4500× *g* using a Sorvall RC-5B centrifuge at 4°C for 10 min, washed in 100 mM potassium phosphate buffer, pH 7.5, pelletted, overlaid with 1 ml of buffer and frozen at −80°C. Cell pellets were thawed and resuspended in 1–5 ml of 100 mM potassium phosphate buffer, pH 7.5 and passed through a French press. Cell extracts were centrifuged at 14,000 rpm for 1–10 min to remove cell debris, depending on the potential oxygen sensitivity of the enzyme to be assayed. A minimum of 2 biological replicates were assayed for the following enzymes: methanol dehydrogenase (MeDH), formate dehydrogenase (Fdh), methylene-H_4_F dehydrogenase (MtdA), β-ketothiolase (βKT), crotonyl-CoA reductase (Ccr), hydroxypyruvate reductase (Hpr), serine-glyoxylate aminotransferase (Sga), malate dehydrogenase (Mdh), aconitase (Acn), succinate dehydrogenase (Sdh), isocitrate dehydrogenase (Icd) and phosphoglucose isomerase (Pgi). βKT, Hpr, Sga and Mdh were measured as previously described [13]. The remaining enzyme activities were measured in a final volume of 200 µL in a Spectramax 190 plate reader (Molecular Devices, Sunnyvale, CA) at room temperature and contained ∼25–100 µg protein. Absorbance data were converted to a 1 cm pathlength by dividing the slope by the conversion factor, 0.541 and protein concentrations were determined spectrophotometrically [41]. With the exception of the 200 µL volume, assays for Fdh [26], MtdA [42], Acn [43], were as described previously. MeDH activity was measured as described [1] except the assay contained 0.4 mM KCN. Ccr activity was measured as described [44] except that 2 mM NADPH was used. Sdh activity was measured as described previously [45] with modifications as described [46]. Icd activity was measured by following the production of NADPH at 340 nm using an extinction coefficient of 6.2 mM^−1^ cm^−1^. The reaction contained 100 mM potassium phosphate buffer, pH 7.5, 1 mM MgCl_2_, 2 mM NADPH, cell extract (∼25–50 µg protein) and was initiated by addition of 20 mM isocitrate. Pgi activity was measured by modification of a coupled assay [47] and followed the production of NADPH at 340 nM in a quartz microtiter plate using an extinction coefficient of 6.2 mM^−1^ cm^−1^. The reaction contained 100 mM potassium phosphate buffer, pH 7.5, 1 mM MgCl_2_, 2 mM NADPH, glucose-6-phosphate dehydrogenase (5 U/ml), cell extract (∼25–50 µg protein) and was initiated by addition of 4 mM fructose-6-phosphate.

### Nucleotide measurements by LC-MS/MS

5 ml of the cell culture was carefully and rapidly pipetted into the center of 25 ml 4-(2-hydroxyethyl)-1-piperazineethanesulfonic acid (HEPES)-buffered (70 mM, pH 6.8) aqueous 60% methanol (v/v) solution (−40°C). The quenched biomass was precipitated in a refrigerated centrifuge (6 min, 10,000 rpm, −20°C; Dupont Sorvall RC5B, Waltham, MA, USA). The supernatant was removed and the cell pellets were resuspended in 5 ml of the same methanol solution and centrifuged for 6 min at 10,000 rpm. For ATP, ADP, AMP, NAD and NADP extraction, 1 ml of boiling HEPES buffered ethanol solution (75% v/v ethanol/water, pH 5.2) was added to a given cell pellet and incubated at 100°C for 3 min [13]. The extracted cell suspension was cooled on ice for 3 min and the cell debris was removed by centrifugation at 5000 rpm for 5 min. The cell-free metabolite extract was again centrifuged at 14,000 rpm for 8 min. The supernatant was transferred into a 2 ml glass vial and dried in a vacuum centrifuge (CentriVap Concentrator System, Labconco, MO, USA). The dried sample, prior to analysis using LC-MS/MS, was re-dissolved in 100 µL purified water for analysis. NADH and NADPH were extracted by a hot alkaline extraction method [13]. 0.8 ml of 0.8 N KOH at 85°C with 1% (v/v) Triton X-100 was added to a given cell pellet, and the mixture was incubated for 3 min at 85°C. After cooling on ice for 3 min, the mixture was centrifuged at 5,000 rpm at 4°C for 5 min. The supernatant was partially neutralized with HClO_4_ to pH 10 and centrifuged at 14,000 rpm for 8 min. The supernatant was analyzed by LC-MS/MS immediately. LC-MS/MS experiments were carried out on a Waters (Milford, MA, USA) LC-MS system consisting of a 1525 µ binary HPLC pump with a 2777C autosampler coupled to a Quattro Micro API triple-quadrupole mass spectrometer (Micromass, Manchester, UK) as described previously [48]. A minimum of two biological replicates with two technical duplicates were measured for each time point.

### CO_2_ assimilation flux

Carbon fluxes for steady-state succinate-limited chemostat cultures were estimated using the dilution rate at steady state (0.163 h^−1^), the dry weight of cells per ml of culture at an OD_600_ of 1.0 (0.278 mg; [21]), and the percentage of total biomass, by weight, that is carbon (47%; [49]). Production of CO_2_ was measured for 2 biological replicates using ^14^C-labeled methanol essentially as described previously [50]; net CO_2_ production was added to CO_2_ assimilation flux estimated at 50% of the carbon in biomass as indicated by the fact that ∼50% of carbon labeling can be eliminated in the presence of high unlabeled CO_2_ as compared with a CO_2_-free environment ([51]; G.J. Crowther, unpublished data). Carbon assimilation into biomass via methylene H_4_F was calculated from changes in OD_600_ using the conversion factors noted above (0.278 mg dry weight per ml of culture at an OD_600_ of 1.0; 47% of biomass is carbon) and also the estimate that 50% of the carbon in biomass comes from assimilated CO_2_.

### CoA and CoA ester measurements by HPLC

CoA and short-chain CoA esters, including acetyl-CoA, malonyl-CoA, methylmalonyl-CoA, 3-hydroxybutyryl-CoA and propionyl-CoA, were measured by reversed-phase high-performance liquid chromatography.

Liquid nitrogen was used to quench metabolism. Extraction was performed by adding 1.5 ml boiling 20% ethanol in 5 mM sodium acetate/acetic acid buffer (pH 3) containing 2 mM DTT to each sample and incubating at 100°C for 3–5 min. Extracted cell suspension was cooled on ice for 3 min and the cell debris was removed by centrifugation at 5,000 rpm for 5 min. The cell-free metabolite extract was dried in a vacuum centrifuge (CentriVap Concentrator system, 79820-00, Labconco, MO, USA) to complete dryness. Deoxy-guanosine was added as an internal standard to correct for volume changes during sample preparation and injection. A Nova-Pac C18 reverse-phase column (3.9×150 mm, 60 Å, 4 µm) from Waters (Milford, MA) was used to separate CoA and CoA esters. A UV detector was used, with the wavelength set to monitor absorbance at 260 nm. Buffer A was composed of 0.1 M sodium dihydrogen phosphate and 0.075 M sodium acetate (pH 4.5); buffer B consisted of 40% buffer A and 60% methanol. The flow rate was 0.8 ml/min and the following HPLC gradient time program was used: 4 min at 100% of buffer A; linearly increase to reach 30% of buffer B at 34 min; increase to 100% buffer B in 7 min; keep at 100% buffer B for 3 min; re-equilibrate the column with buffer A for 6 min. Hydroxylamine reacts with CoA esters to form CoA and the corresponding free acids, and this reaction was used to confirm CoA compounds. Neutral hydroxylamine solution was prepared by adjusting NH_2_OH-HCl solution with 10 N NaOH to pH 7. To three volumes of an analyte solution, one volume of 2 M neutral NH_2_OH solution was added. The mixture was incubated at 30°C for approximately 1 hour to achieve complete conversion of CoA esters to CoA.

These CoA compounds were measured from two biological replicate samples taken at 0 min (before methanol addition), 10 min, 30 min, 1 h, and 3 h after methanol addition. Standard curves were generated for quantitation of the CoA compounds by adding known amounts to cell extracts (data not shown). Recoveries of CoA and CoA esters under the extraction condition were determined by spiking known amounts of standard CoA compounds to a cell culture sample before quenching and extraction. Recoveries ranged from 70–100%, depending on the compound.

### GC×GC-TOFMS analysis of cell extracts

Two-dimensional gas chromatography coupled with time-of-flight mass spectrometry was used to analyze cell extract samples for the measurement of amino acids, organic acids and carbohydrates. Samples were taken at 0 min (before methanol addition), 10 min, 20 min, 30 min, 1 h, 2 h and 3 h after methanol addition, and were analyzed by GC×GC-TOFMS as described [1]. Numbers are averages of nine replicates (three extractions with 3 biological replicate injections for each extraction).

### Fisher Ratio analysis of cell extracts

Fisher ratio analysis was used to assess metabolite differences between two conditions as previously described [15]. Numbers are averages of nine replicates (three extractions with 3 biological replicate injections for each extraction).

## Supporting Information

Supplementary Figure S1 Pathway schematics depicting changes that occurred in measured metabolites, gene expression, and enzymatic activities for central metabolism after cells were transitioned from succinate to methanol growth. A boxed gene/protein name indicates the activity of this enzyme was measured. Red lettering for the protein designation indicates an increase in activity; green, decrease; black, no change. Metabolites appearing more than once are connected by gray lines. Diagrams are shown for the following time periods: (A) pre-methanol addition, time  = 0 min; (B) initial response, time  = 10–30 min post methanol addition; (C) just prior to/at the start of cell growth, time  = 1–2 h post methanol addition; and (D) exponential cell growth, time  = 3–6 h post methanol addition with (E) serving as a legend. Gene expression intensities are represented by arrow thickness while gene expression fold changes are depicted by arrowhead number. Abbreviations and reaction descriptions are included in Supplementary Table II along with gene expression intensities, LogRatios, fold changes and p-values. Mesaconyl-CoA, ethylmalonyl-CoA, methylsuccinyl-CoA were measured as free acids. **Color at T = 0 represents the concentration before methanol was added for all metabolites except for methanol which was calculated as 50 mM for the initial T = 0 value.
(0.21 MB PDF)

Supplementary Table S1 Gene expression data for cellular processes that significantly changed during the transition from succinate to methanol growth.
(0.96 MB XLS)

Supplementary Table S2 Gene expression data for central metabolic pathways during the transition from succinate to methanol growth.
(0.40 MB XLS)
