# Supplementary material for: A Systems Biology Approach Uncovers Cellular Strategies Used by Methylobacterium extorquens AM1 During the Switch from Multi- to Single-Carbon Growth
Source: PLoS One. 2010 Nov 24;5(11):e14091. doi: 10.1371/journal.pone.0014091 (PMC2991311; doi:10.1371/journal.pone.0014091)
Supplement: Supplementary Figure S1 — Pathway schematics depicting changes that occurred in measured metabolites, gene expression, and enzymatic activities for central metabolism after cells were transitioned from succinate to methanol growth. A boxed gene/protein name indicates the activity of this enzyme was measured. Red lettering for the protein designation indicates an increase in activity; green, decrease; black, no change. Metabolites appearing more than once are connected by gray lines. Diagrams are shown for the following time periods: (A) pre-methanol addition, time = 0 min; (B) initial response, time = 10–30 min post methanol addition; (C) just prior to/at the start of cell growth, time = 1–2 h post methanol addition; and (D) exponential cell growth, time = 3–6 h post methanol addition with (E) serving as a legend. Gene expression intensities are represented by arrow thickness while gene expression fold changes are depicted by arrowhead number. Abbreviations and reaction descriptions are included in Supplementary Table II along with gene expression intensities, LogRatios, fold changes and p-values. Mesaconyl-CoA, ethylmalonyl-CoA, methylsuccinyl-CoA were measured as free acids. **Color at T = 0 represents the concentration before methanol was added for all metabolites except for methanol which was calculated as 50 mM for the initial T = 0 value. (0.21 MB PDF) [file pone.0014091.s001.pdf]

A

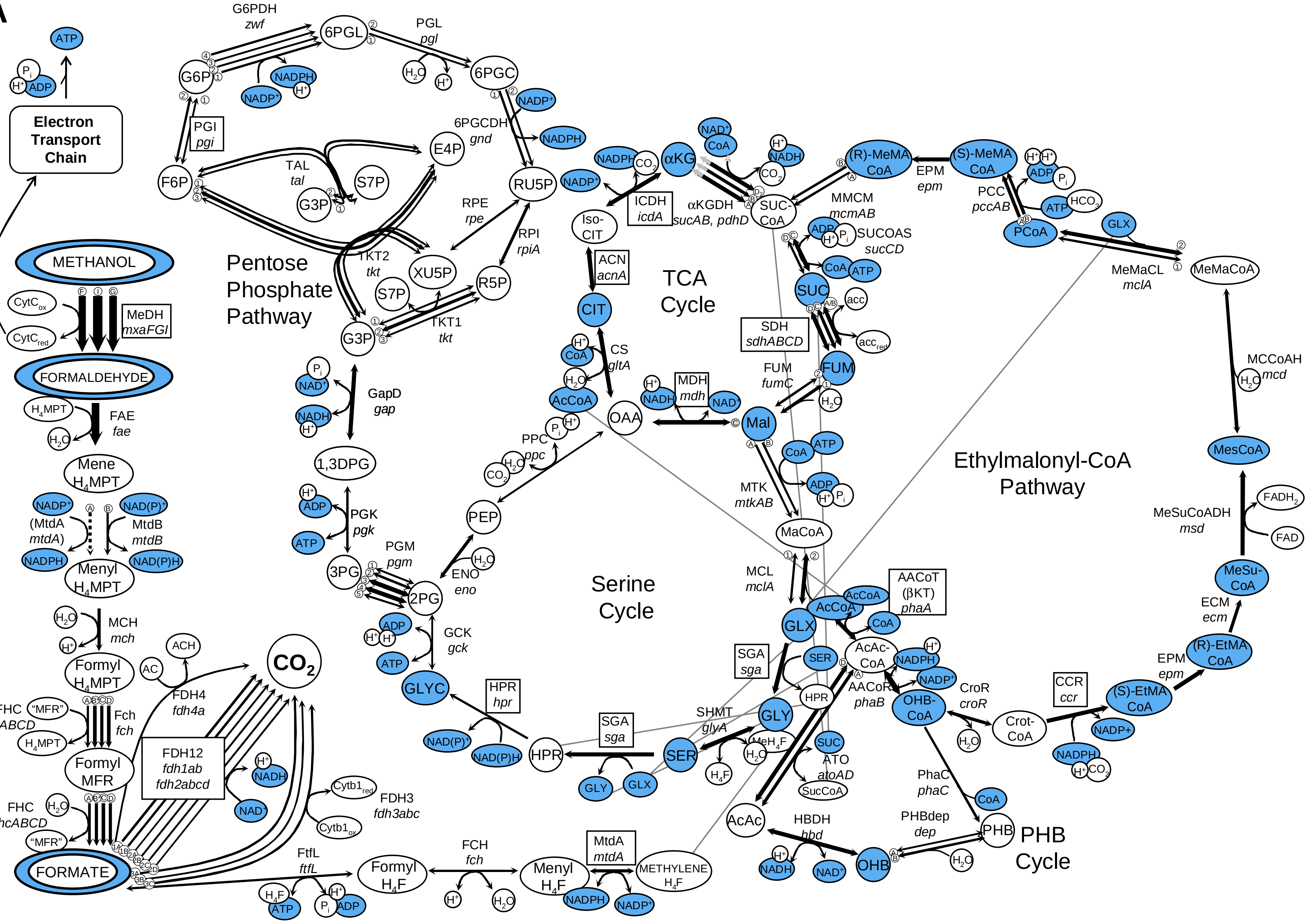

Electron Transport Chain
ATP
METHANOL
MeDH
mxaFGI
FORMALDEHYDE
FAE
fae
Mene H4MPT
(MtdA
mtdA)
MtdB
mtdB
Menyl H4MPT
MCH
mch
Formyl H4MPT
FHC
fhcABCD
Fch
fch
Formyl MFR
FDH4
fdh4a
FDH12
fdh1ab
fdh2abcd
FDH3
fdh3abc
FORMATE
CO2
FtfL
ftfL
Formyl H4F
FCH
fch
Menyl H4F
MtdA
mtdA
METHYLENE H4F
G6PDH
zwf
6PGL
PGL
pgl
6PGC
G6P
PGI
pgi
F6P
6PGCDH
gnd
RU5P
E4P
TAL
tal
S7P
G3P
RPE
rpe
RPI
rpiA
TKT2
tkt
XU5P
R5P
TKT1
tkt
Pentose Phosphate Pathway
GapD
gap
1,3DPG
PGK
pgk
3PG
PGM
pgm
2PG
ENO
eno
PEP
PPC
ppc
GCK
gck
GLYC
HPR
hpr
SGA
sga
TCA Cycle
αKG
ICDH
icdA
Iso-CIT
ACN
acnA
CIT
CS
gltA
AcCoA
OAA
MDH
mdh
Mal
FUM
fumC
SDH
sdhABCD
SUC
SUCOAS
sucCD
SUC-CoA
αKGDH
sucAB, pdhD
MMCM
mcmAB
MTK
mtkAB
MaCoA
MCL
mclA
Serine Cycle
GLX
SHMT
glyA
GLY
SER
HPR
AACoT (βKT)
phaA
AcAc-CoA
AACoR
phaB
ATO
atoAD
AcAc
HBDH
bbd
OHB
PhaC
phaC
PHBdep
dep
PHB
PHB Cycle
(R)-MeMA CoA
EPM
epm
(S)-MeMA CoA
PCC
pccAB
PCoA
MeMaCL
mclA
MeMaCoA
MCCoAH
mcd
MesCoA
MeSuCoADH
msd
MeSu-CoA
ECM
ecm
(R)-EtMA CoA
(S)-EtMA CoA
CCR
ccr
Crot-CoA
CroR
croR
OHB-CoA
Ethylmalonyl-CoA Pathway

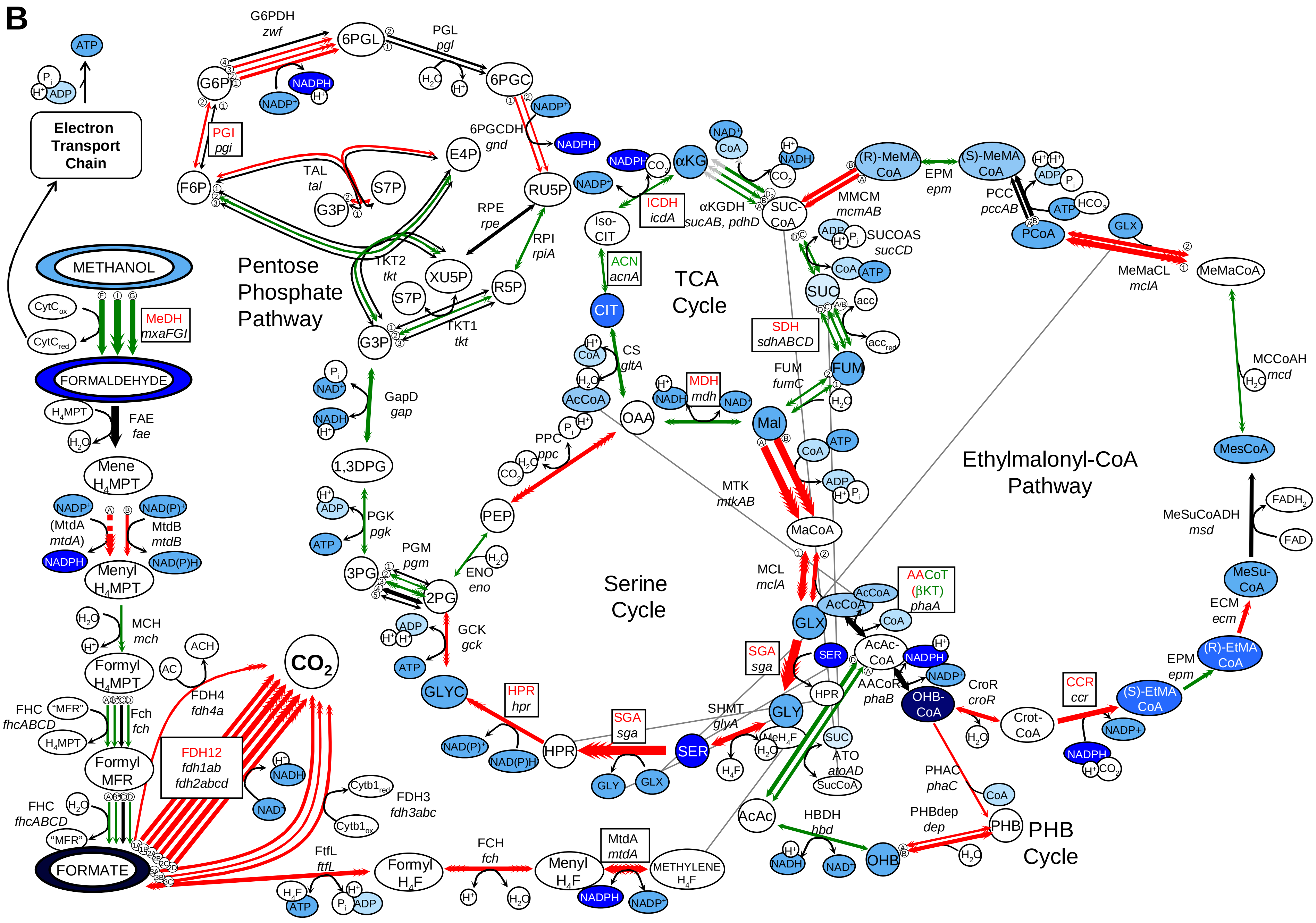
B
ATP
Electron Transport Chain
METHANOL
CytC$_{ox}$
CytC$_{red}$
MeDH
mxaFGI
FORMALDEHYDE
H_4MPT
FAE
fae
H_2O
Mene H_4MPT
$NADP^+$
$NAD(P)^+$
(MtdA
mtdA)
MtdB
mtdB
NADPH
NAD(P)H
Menyl H_4MPT
MCH
mch
Formyl H_4MPT
FHC
fhcABCD
"MFR"
Fch
fch
Formyl MFR
FDH12
fdh1ab
fdh2abcd
FDH4
fdh4a
ACH
AC
NADH
NAD^+
Cytb1$_{red}$
Cytb1$_{ox}$
FDH3
fdh3abc
CO_2
FORMATE
FtfL
ftfL
Formyl H_4F
FCH
fch
Menyl H_4F
MtdA
mtdA
METHYLENE H_4F
G6PDH
zwf
6PGL
PGL
pgl
6PGC
G6P
PGI
pgi
F6P
6PGCDH
gnd
RU5P
E4P
TAL
tal
S7P
G3P
RPE
rpe
RPI
rpiA
Pentose Phosphate Pathway
TKT2
tkt
XU5P
R5P
TKT1
tkt
GapD
gap
1,3DPG
PGK
pgk
3PG
PGM
pgm
2PG
ENO
eno
PEP
PPC
ppc
GCK
gck
GLYC
HPR
hpr
NAD(P)$^+$
NAD(P)H
SGA
sga
GLY
GLX
SER
SHMT
glyA
MeH$_4$F
H_4F
Serine Cycle
αKG
ICDH
icdA
Iso-CIT
ACN
acnA
CIT
CS
gltA
AcCoA
OAA
MDH
mdh
Mal
TCA Cycle
αKGDH
sucAB, pdhD
SUC-CoA
SUCOAS
sucCD
SUC
acc
acc$_{red}$
SDH
sdhABCD
FUM
FUM
fumC
MTK
mtkAB
MaCoA
MCL
mclA
AACoT
(βKT)
phaA
AcAc-CoA
AACoR
phaB
OHB-CoA
HPR
ATO
atoAD
SucCoA
AcAc
HBDH
hbd
OHB
PHAC
phaC
PHBdep
dep
PHB
PHB Cycle
(R)-MeMA CoA
EPM
epm
(S)-MeMA CoA
MMCM
mcmAB
PCC
pccAB
PCoA
HCO_3
MeMaCL
mclA
MeMaCoA
MCCoAH
mcd
Ethylmalonyl-CoA Pathway
MesCoA
MeSuCoADH
msd
$FADH_2$
FAD
MeSu-CoA
ECM
ecm
(R)-EtMA CoA
EPM
epm
(S)-EtMA CoA
CCR
ccr
CroR
croR
Crot-CoA
NADP+

C

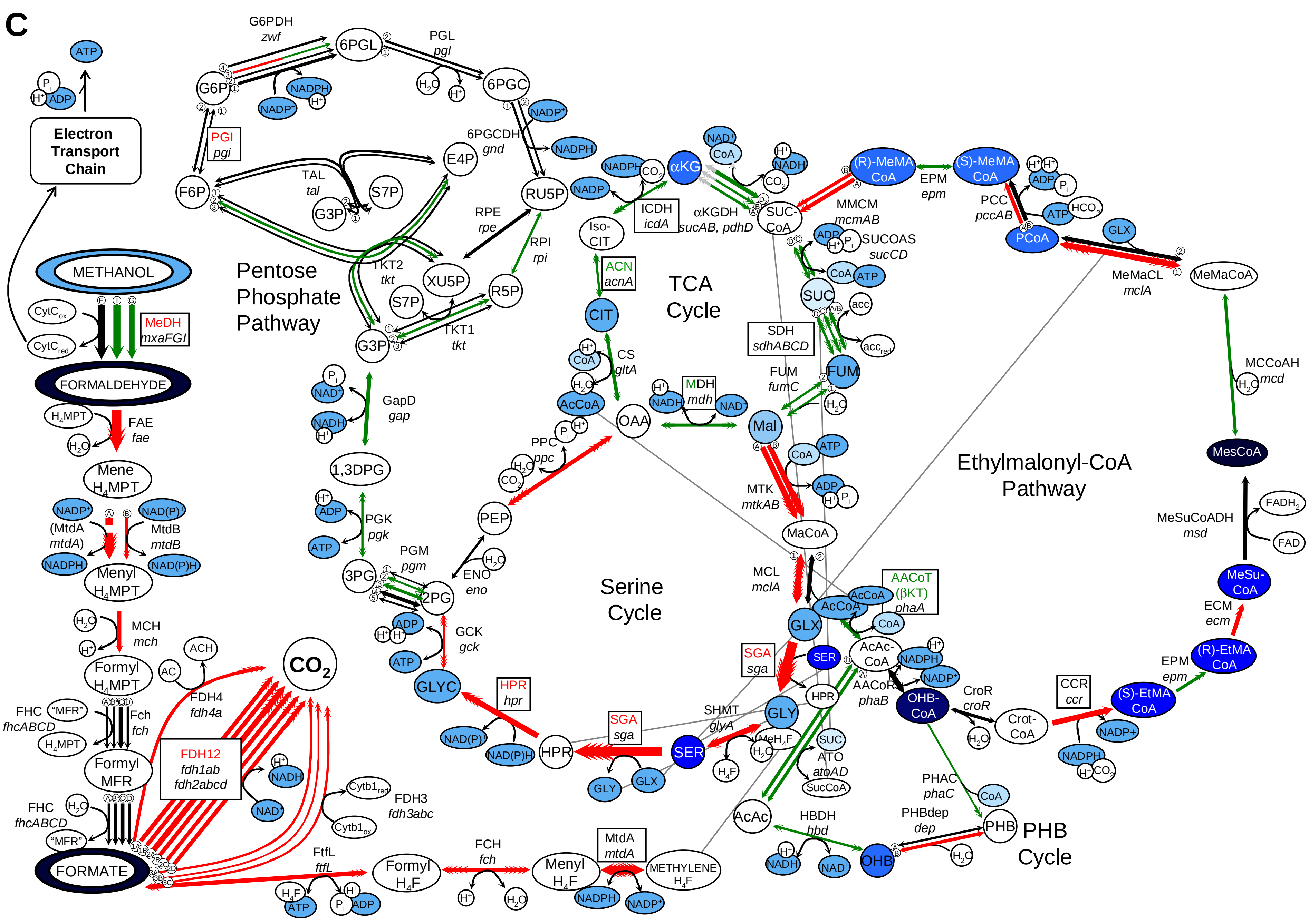

ATP
ADP
Electron Transport Chain
METHANOL
CytC_ox
CytC_red
MeDH
mxaFGI
FORMALDEHYDE
H4MPT
H2O
FAE
fae
Mene H4MPT
NADP+
NAD(P)+
(MtdA
mtdA)
MtdB
mtdB
NADPH
NAD(P)H
Menyl H4MPT
MCH
mch
Formyl H4MPT
AC
ACH
FDH4
fdh4a
FHC
fhcABCD
"MFR"
Fch
fch
Formyl MFR
FDH12
fdh1ab
fdh2abcd
NADH
NAD+
CO2
Cytb1red
Cytb1ox
FDH3
fdh3abc
FORMATE
FtfL
ftfL
H4F
Pi
Formyl H4F
FCH
fch
H+
Menyl H4F
MtdA
mtdA
METHYLENE H4F
G6PDH
zwf
6PGL
PGL
pgl
G6P
NADPH
6PGC
6PGCDH
gnd
PGI
pgi
F6P
TAL
tal
E4P
S7P
G3P
RU5P
RPE
rpe
RPI
rpi
TKT2
tkt
XU5P
R5P
TKT1
tkt
Pentose Phosphate Pathway
GapD
gap
1,3DPG
PGK
pgk
3PG
PGM
pgm
2PG
ENO
eno
PEP
PPC
ppc
GCK
gck
GLYC
HPR
hpr
NAD(P)+
NAD(P)H
SGA
sga
GLY
GLX
SER
SHMT
glyA
MeH4F
Serine Cycle
OAA
CS
gltA
AcCoA
CoA
CIT
ACN
acnA
Iso-CIT
ICDH
icdA
αKG
αKGDH
sucAB, pdhD
SUC-CoA
TCA Cycle
MDH
mdh
SDH
sdhABCD
SUCOAS
sucCD
SUC
acc
acc_red
FUM
FUM
fumC
Mal
MTK
mtkAB
MaCoA
MCL
mclA
AACoT
(βKT)
phaA
AcAc-CoA
AACoR
phaB
HPR
ATO
atoAD
SucCoA
AcAc
HBDH
hbd
OHB
PHAC
phaC
PHBdep
dep
PHB
PHB Cycle
(R)-MeMA CoA
(S)-MeMA CoA
EPM
epm
MMCM
mcmAB
PCC
pccAB
HCO3
PCoA
MeMaCL
mclA
MeMaCoA
MCCOAH
mcd
MesCoA
Ethylmalonyl-CoA Pathway
MeSuCoADH
msd
FADH2
FAD
MeSu-CoA
ECM
ecm
(R)-EtMA CoA
(S)-EtMA CoA
CCR
ccr
Crot-CoA
CroR
croR
OHB-CoA

D

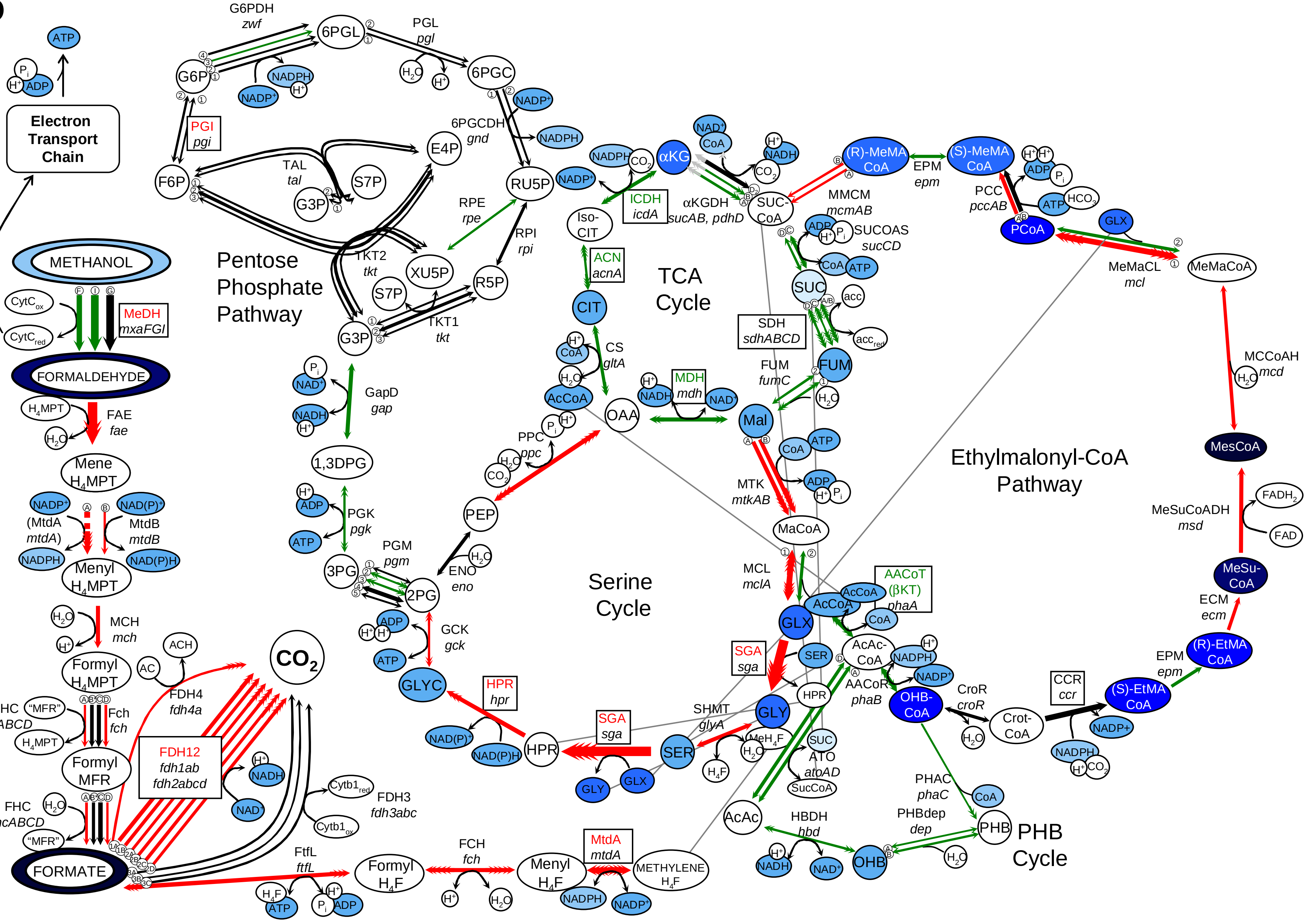

ATP
P_i
H^+ ADP
Electron Transport Chain
METHANOL
$CytC_{ox}$
$CytC_{red}$
F I G
MeDH
mxaFGI
FORMALDEHYDE
H_4MPT
FAE
fae
H_2O
Mene H_4MPT
$NADP^+$
$NAD(P)^+$
A B
(MtdA
mtdA)
MtdB
mtdB
NADPH
NAD(P)H
Menyl H_4MPT
H_2O
MCH
mch
H^+
ACH
AC
Formyl H_4MPT
FDH4
fdh4a
FHC
fhcABCD
"MFR"
Fch
fch
H_4MPT
Formyl MFR
FDH12
fdh1ab
fdh2abcd
H^+
NADH
NAD^+
FHC
fhcABCD
H_2O
"MFR"
FORMATE
CO_2
$Cytb1_{red}$
FDH3
fdh3abc
$Cytb1_{ox}$
FtfL
ftfL
H_4F
ATP
P_i
H^+
ADP
Formyl H_4F
FCH
fch
H^+
H_2O
Menyl H_4F
MtdA
mtdA
NADPH
$NADP^+$
METHYLENE H_4F
G6PDH
zwf
6PGL
PGL
pgl
G6P
NADPH
$NADP^+$
H^+
H_2O
H^+
6PGC
$NADP^+$
6PGCDH
gnd
NADPH
PGI
pgi
F6P
TAL
tal
E4P
S7P
G3P
RU5P
RPE
rpe
RPI
rpi
TKT2
tkt
XU5P
S7P
R5P
G3P
TKT1
tkt
Pentose Phosphate Pathway
P_i
NAD^+
NADH
H^+
GapD
gap
1,3DPG
H^+
ADP
PGK
pgk
ATP
3PG
PGM
pgm
2PG
ENO
eno
H_2O
PEP
PPC
ppc
P_i
H^+
H_2O
CO_2
ADP
H^+ H^+
ATP
GCK
gck
GLYC
HPR
hpr
$NAD(P)^+$
NAD(P)H
HPR
SGA
sga
GLY
GLX
SER
Serine Cycle
SHMT
glyA
MeH_4F
H_2O
H_4F
NADPH
CO_2
αKG
NAD^+
CoA
H^+
NADH
CO_2
$NADP^+$
ICDH
icdA
αKGDH
sucAB, pdhD
SUC-CoA
Iso-CIT
ACN
acnA
CIT
TCA Cycle
H^+
CoA
CS
gltA
H_2O
AcCoA
OAA
H^+
NADH
MDH
mdh
NAD^+
Mal
SDH
sdhABCD
FUM
fumC
FUM
H_2O
MMCM
mcmAB
ADP
P_i
H^+
SUCOAS
sucCD
CoA
ATP
SUC
acc
acc_{red}
CoA
ATP
ADP
H^+ P_i
MTK
mtkAB
MaCoA
MCL
mclA
GLX
AcCoA
AcCoA
AACoT
(βKT)
phaA
CoA
SGA
sga
SER
HPR
GLY
AcAc-CoA
NADPH
H^+
$NADP^+$
AACoR
phaB
SUC
ATO
atoAD
SucCoA
AcAc
HBDH
hbd
H^+
NADH
NAD^+
OHB
(R)-MeMA CoA
EPM
epm
(S)-MeMA CoA
H^+ H^+
ADP
P_i
PCC
pccAB
ATP
HCO_3
PCoA
GLX
MeMaCL
mcl
MeMaCoA
MCCoAH
mcd
H_2O
MesCoA
Ethylmalonyl-CoA Pathway
$FADH_2$
MeSuCoADH
msd
FAD
MeSu-CoA
ECM
ecm
EPM
epm
(R)-EtMA CoA
(S)-EtMA CoA
CroR
croR
CCR
ccr
OHB-CoA
H_2O
Crot-CoA
$NADP+$
NADPH
H^+ CO_2
PHAC
phaC
CoA
PHBdep
dep
PHB
H_2O
PHB Cycle

E

PATHWAY KEY

ARROWS:

Thickness	Processed Signal Intensity
→	0-500
→	501-1000
→	1001-5000
→	5001-10,000
→	10,001-50,000
→	50,01-100,000
→	100,001-200,000
→	>200,000

Arrowhead No.	Fold Change
1	1.5-1.9
2	2-4.9
3	5-9.9
4	10-19.9
5	20-49.9
6	50-99.9
7	≥100

Arrow Color	Description
Red	Increase in expression
Green	Decrease in expression
Black	No change in expression

METABOLITES:

Color	Fold Change/Description
	>10x increase
	5.1-10x increase
	2.1-5x increase
	1.5-2x increase
	Concentration at T=0**
	1.5-2x decrease
	2.1-5x decrease
	>5x decrease
	Was not measured

Pattern	Measurement Type
○	Intracellular
◎	Extracellular/supernatant

Symbols:

(1) Number designates gene number if multiple genes per reaction

(A) Letter designates gene name or subunit label

Supplementary figure S1. Pathway schematics depicting changes that occurred in measured metabolites, gene expression, and enzymatic activities for central metabolism after cells were transitioned from succinate to methanol growth. A boxed gene/protein name indicates the activity of this enzyme was measured. Red lettering for the protein designation indicates an increase in activity; green, decrease; black, no change. Metabolites appearing more than once are connected by gray lines. Diagrams are shown for the following time periods: (A) pre-methanol addition, time = 0 min; (B) initial response, time = 10-30 min post methanol addition; (C) just prior to/at the start of cell growth, time = 1-2 h post methanol addition; and (D) exponential cell growth, time = 3-6 h post methanol addition with (E) serving as a legend. Gene expression intensities are represented by arrow thickness while gene expression fold changes are depicted by arrowhead number. Abbreviations and reaction descriptions are included in Supplementary Table II along with gene expression intensities, LogRatios, fold changes and p-values. Mesaconyl-CoA, ethylmalonyl-CoA, methylsuccinyl-CoA were measured as free acids. **Color at T=0 represents the concentration before methanol was added for all metabolites except for methanol which was calculated as 50 mM for the initial T=0 value.
